# Supplementary material for: Respiratory viral infections awaken metastatic breast cancer cells in lungs
Source: Nature. 2025 Jul 30;645(8080):496–506. doi: 10.1038/s41586-025-09332-0 (PMC12422975; doi:10.1038/s41586-025-09332-0)
Supplement: Supplementary file 1 — Supplementary Information [file 41586_2025_9332_MOESM1_ESM.pdf]

nature portfolio

https://doi.org/10.1038/s41586-025-09332-0

Supplementary information

Respiratory viral infections awaken metastatic breast cancer cells in lungs

In the format provided by the authors and unedited

Supplemental Note 1.

Recent studies have shown that respiratory viruses, including SARS-CoV-2, can significantly impair mitochondrial function, resulting in persistent immune dysregulation post-infection[1,2]. Here, we demonstrate that influenza A virus (IAV) infection disrupts mitochondrial function in immune cells from BALF of mice up to 15 days post-infection, as evidenced by reduced mitochondrial content (decreased MitoTracker staining; **Extended Data Figure 7g**) and widespread transcriptional alterations (**Extended Data Figures 7a and 8**).

At 15 days post-infection, immune cells from the lungs of MMTV-Her2 and WT mice exhibited significant changes in oxidative phosphorylation (OXPHOS) and mitochondrial metabolism, likely contributing to immune dysfunction that hinders the clearance of Her2+ disseminated cancer cells (DCC) (**Extended Data Figure 7a and 8**).

Across multiple T cell populations, there was consistent downregulation of mitochondrial DNA (mtDNA)-encoded OXPHOS transcripts, except for *mt-Nd6* (NADH dehydrogenase subunit 6), which remained unchanged or upregulated (**Extended Data Figure 8**). This resilience may stem from mt-Nd6 being transcribed from the mtDNA light strand, unlike other mitochondrial genes transcribed from the heavy strand.

Nuclear-encoded OXPHOS transcripts were also generally repressed, particularly for Complexes I and V of the electron transport chain. With the notable exception of Complex II genes, for which there is a selective upregulation, including succinate dehydrogenase complex flavoprotein subunit A (*Sdha*), B (*Sdhb*), C (*Sdhc*), D (*Sdhd*), and *Sdhaf1* (succinate dehydrogenase assembly factor 1) across various cell types (**Extended Data Figure 8**). This upregulation of Complex II genes, encoded entirely by nuclear DNA, presumably is a compensatory response to mtDNA dysfunction, helping to preserve some mitochondrial function.

Additional indicators of mitochondrial dysfunction include the consistent downregulation of *Slc25a5* (a solute carrier protein critical for adenine nucleotide transport across the mitochondrial membrane) and *Pkm* (pyruvate kinase M, a key enzyme bridging cytoplasmic glycolysis and mitochondrial metabolism). Together with OXPHOS repression, this highlights a severe disruption in cellular energy production, indicative of broad metabolic collapse. Conversely, *Cpt1a*, encoding the rate-limiting enzyme for fatty acid oxidation (FAO), was significantly upregulated (**Extended Data Figure 8**). Its transcriptional activation in low-energy states underscores an adaptive mechanism to sustain energy production under metabolic stress.

In B cells and effector T cells, mitochondrial dysfunction and reduced energy production coincided with immune dysregulation. This included downregulation of cytokine and interleukin pathways and deregulation of innate and adaptive immune genes in macrophages, complement macrophages, and inflammatory monocytes, impairing their ability to perform effective immune surveillance against tumor cells (**Extended Data Figure 7a**). At the individual gene level, immune regulators such as *Cxcl10* (C-X-C motif chemokine ligand 10), *Zbp1* (Z-DNA binding protein 1), and *Tnf* were consistently downregulated across multiple cell types, further reducing the capacity to detect and eliminate tumor cells (**Extended Data Figure 7a**).

Anti-CD4 treatment in MMTV-Her2 mice effectively reversed these metabolic effects by 15 days post infection in $CD8^+$ cells. At 15 days, we observe robust upregulation of mtDNA-encoded OXPHOS transcripts and *Slc25a5*, alongside decreased *Cpt1a* expression (**Extended Data Figure 8**). This metabolic recovery correlated with consistently elevated immune transcripts at both time points, indicating enhanced immune surveillance (**Extended Data Figure 7a**).

These findings reveal that IAV infection triggers mitochondrial dysfunction and bioenergetic decline in immune cells, characterized by downregulated OXPHOS pathways, diminished mitochondrial content, and disrupted metabolic processes. This decline is associated with reduced immune gene expression, fostering an immunosuppressive environment conducive to tumor progression. Additionally, the results suggest that IAV triggers the production of a factor in $CD4^+$ cells that suppresses mitochondrial and immune functions in $CD8^+$ cells, delaying their recovery. Importantly, anti-CD4 treatment counteracted this effect, accelerating both metabolic and immune recovery, enhancing immune surveillance, and creating conditions supportive of tumor suppression.

Citations

1 Guarnieri, J. W. *et al.* Core mitochondrial genes are down-regulated during SARS-CoV-2 infection of rodent and human hosts. *Sci Transl Med* **15**, eabq1533 (2023). https://doi.org:10.1126/scitranslmed.abq1533

2 Guarnieri, J. W. *et al.* Lethal COVID-19 Associates With RAAS-Induced Inflammation For Multiple Organ Damage Including Mediastinal Lymph Nodes. *bioRxiv*, 2023.2010.2008.561395 (2023). https://doi.org:10.1101/2023.10.08.561395 (*in press, PNAS*)

Extended Data Figure 7b – Western blot

In figure:

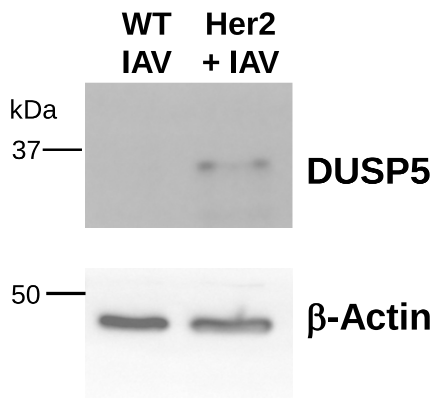

Cropped:

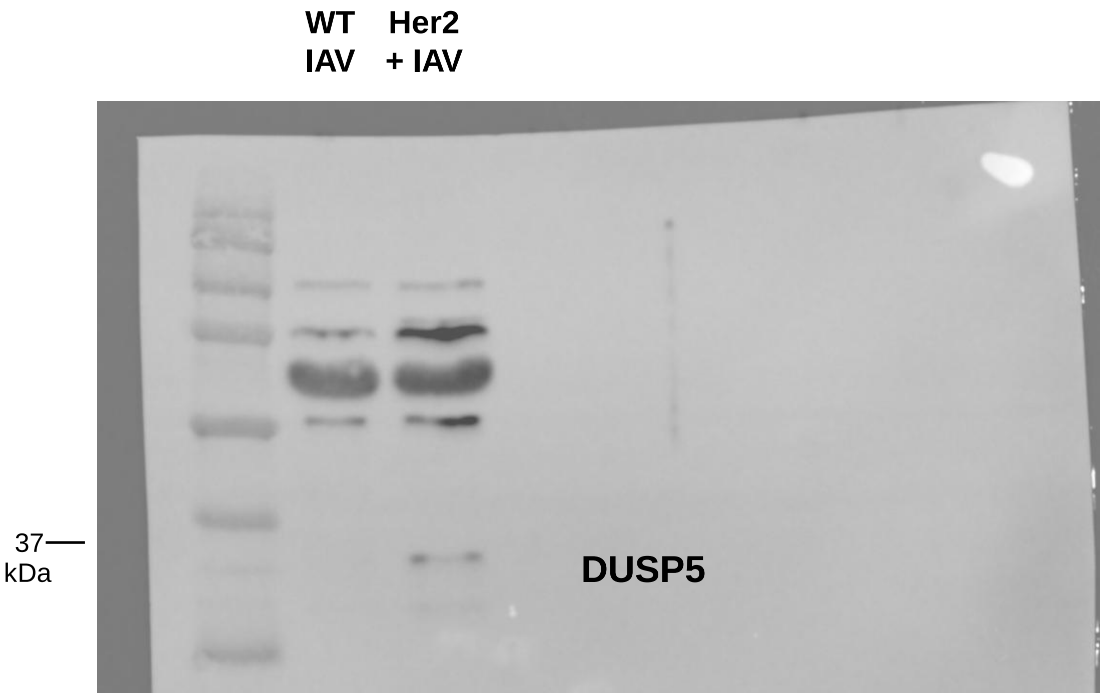

DUSP5 and b-Actin westerns were from the same gel

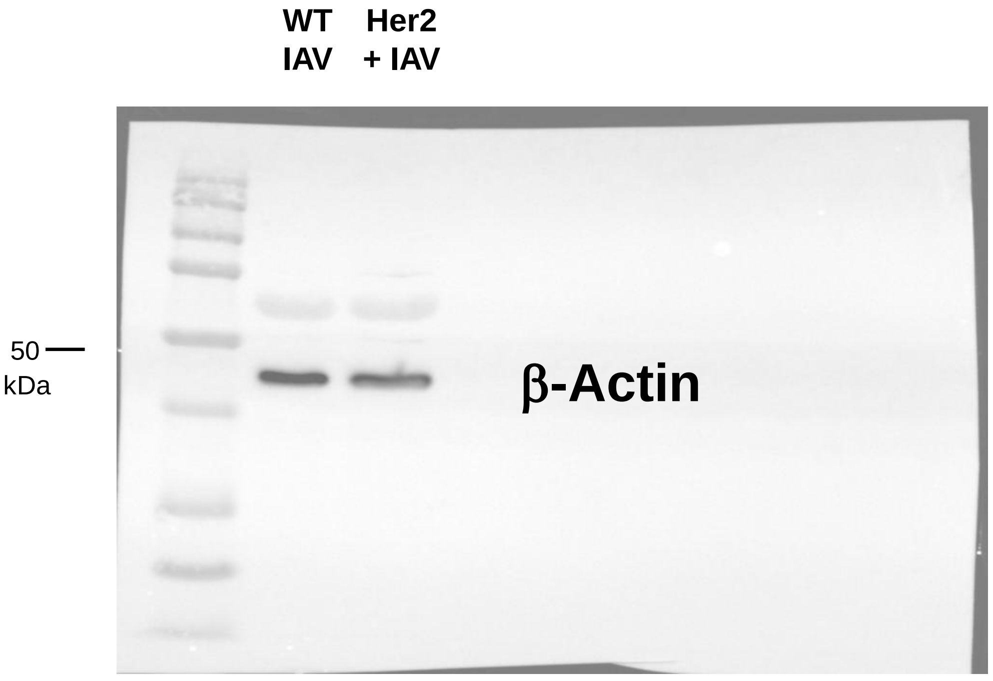

Supplementary Figure 1

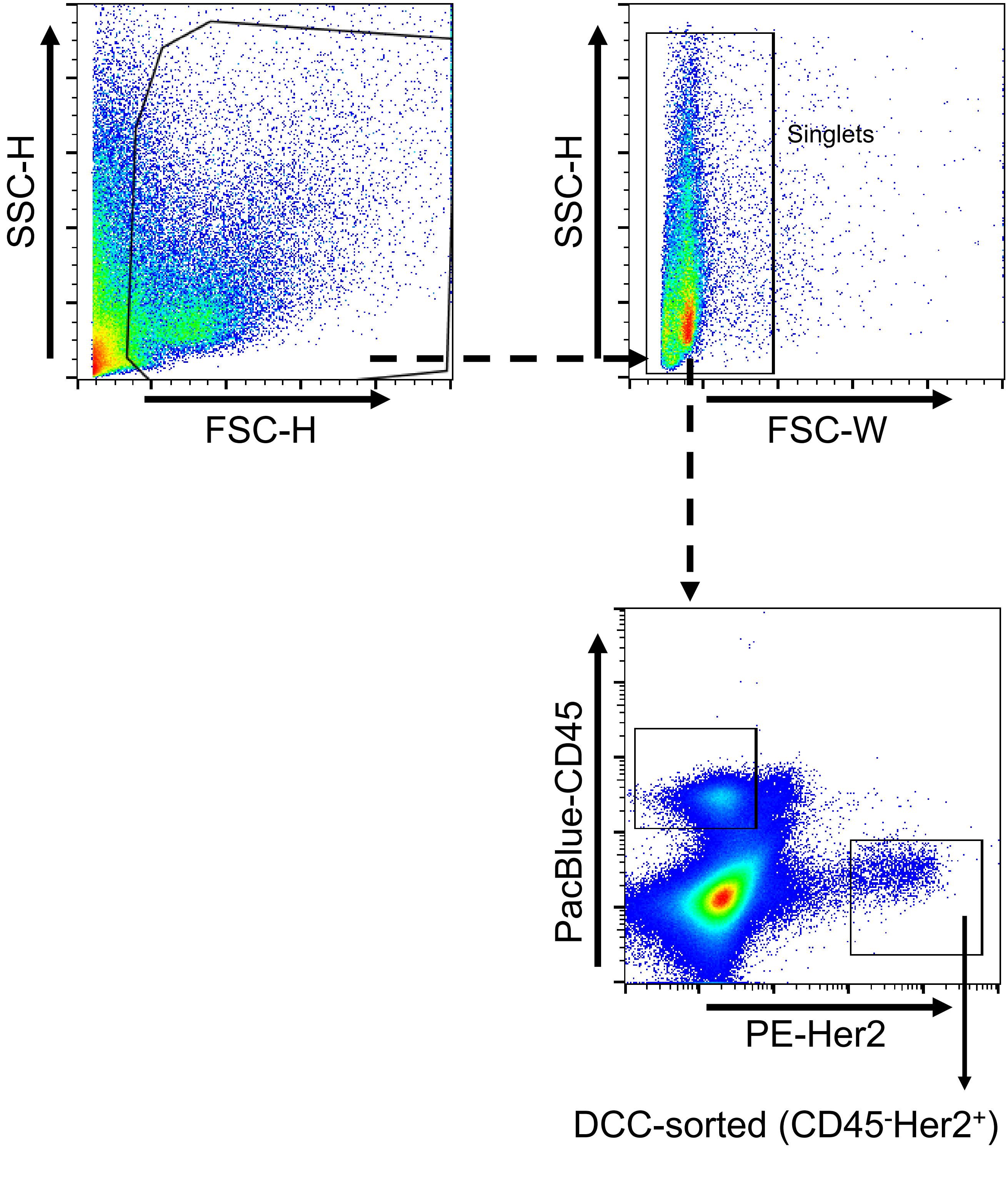

Supplementary Figure 2: Gating strategies for DCC sorting (used in Figure 2e-h). DCC were sorted based on SSC/FSC, singlets, CD45 negative and Her2 positive.

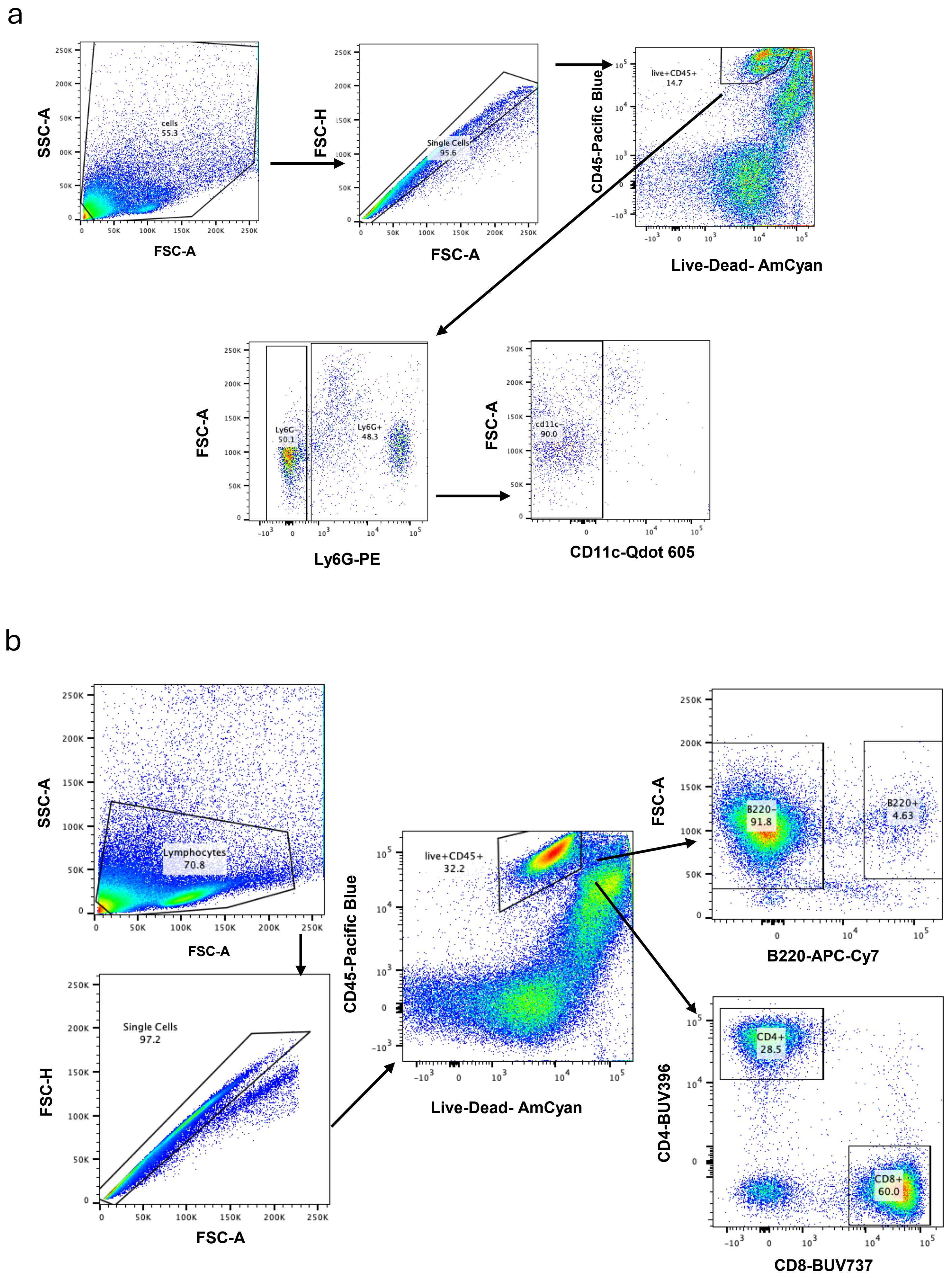

Supplementary Figure 3: Gating strategies for EDF-4. Gating strategy for neutrophils shown in EDF4a (a). Gating strategy for CD4+,CD8+ and B220+ cells shown in EDFb-d (b)

Supplemental Information Table 1: Resource table

REAGENT OR RESOURCE	SOURCE	IDENTIFIER	Dilution
Antibodies - Immunohistochemistry			
Anti-c-ErbB2/c-Neu (Ab-4) Mouse mAb (7.16.4) (0.1 mg/mL)	Millipore	Cat # OP16; RRID: AB_2099539	1:100
Anti-HER2/ErbB2 (29D8) Rabbit mAb	Cell Signaling Technology	Cat # 2165; RRID: AB_10692490	1:100
Rabbit mAb [SP6] to Ki67	Abcam	Cat # ab16667; RRID: AB_302459	1:200
Vimentin (D21H3) XP® Rabbit mAb	Cell Signaling Technology	Cat # 5741; RRID: AB_10695459	1:200
Anti-Mouse CD45R (B220) Rat mAb, Clone RA3-6B2	Stem Cell Technologies	Cat # 60019; RRID: AB_627078	1:200
Recombinant Anti-CD8 alpha rabbit mAb [CAL38]	Abcam	Cat # ab237723; RRID: AB_2864723	1:250
Anti-CD4 (4SM95) rat mAb	Invitrogen	Cat # 14-9766-82; RRID: AB_2573008	1:250
Anti-CD326 (EpCAM) Rat mAb(G8.8)	Invitrogen	Cat # 14-5791-81; RRID: AB_953624	1:50
Anti-CD45 rabbit pAb	Abcam	Cat # ab10558; RRID: AB_442810	1:250
Anti- Cytokeratin 8 Recombinant Rabbit mAb (SU0338)	Invitrogen	Cat # MA5-32118; RRID: AB_2809410	1:200
Anti-GL7 Rat mAb	Invitrogen	Cat # 14-5902-82 RRID: AB_467715	1:250
Recombinant Anti-Myeloperoxidase rabbit mAb [EPR20257]	Abcam	Cat # ab208670; RRID: AB_2864724	1:200
Goat anti-Mouse IgG (H+L) Alexa Fluor™ Plus 555	Invitrogen	Cat # A32727; RRID: AB_2633276	1:500
Goat anti-Rabbit IgG (H+L) Alexa Fluor™ Plus 647	Invitrogen	Cat # A32733; RRID: AB_2633282	1:500
Goat anti-Rat IgG (H+L) Alexa Fluor™ 647	Invitrogen	Cat # A-21247; RRID: AB_141778	1:500
Goat anti-Rat IgG (H+L) Alexa Fluor™ 488	Invitrogen	Cat # A-11006; RRID: AB_141373	1:500
Goat anti-Rabbit IgG (H+L) Alexa Fluor™ 555	Invitrogen	Cat # A-21428; RRID: AB_141784	1:500
Antibodies – Flow Cytometry			
CD45-Pacific Blue Clone 30-F11	Biolegend	Cat # 103126 PRID: AB_493535	1:200
CD45-BV785 Clone 30-F11	Biolegend	Cat # 103149 RRID: AB_2564590	1:200
Ly6G-PE Clone 1A8	Biolegend	Cat # 127608 PRID: AB_1186099	1:200
CD44-APC/Fire750 Clone NIM-R8	Biolegend	Cat # 156003 PRID: AB_2910319	1:100

CD4-BUV395 Clone GK1.5	BD Biosciences	Cat # 563790<br>RRID: AB_2738426	1:200
CD4-BUV737 Clone RM4-5	BD Biosciences	Cat # 612844	1:200
CD4-Pacific Blue Clone RM4-5	Biolegend	Cat #100531<br>RRID: AB_493374	1:200
CD8α-BUV737 Clone 53-6.7	BD Biosciences	Cat # 612759<br>RRID: AB_2870090	1:200
CD8α-BUV395 Clone 53-6.7	BD Biosciences	Cat # 565968<br>RRID: AB_2732919	1:200
B220-APC/Cy7 Clone RA3-6B2	Biolegend	Cat # 103224<br>PRID: AB_313007	1:200
CXCR4-BV421 Clone L276F12	Biolegend	Cat # 146511<br>RRID: AB_2562788	1:200
ErbB2/Her2 Antibody Clone HRB2/258	Novusbio	Cat # NBP2-34641PE	1:100
Antibodies – Western Blot			
β-actin Clone AC-15	Invitrogen	Cat # AM4302	1:1000
DUSP5	Invitrogen	Cat # PA5-85961<br>RRID: AB_2802762	1:1000
Anti-rabbit HRP	Jackson ImmunoResearch Laboratories	Cat # 111-035-144	1:4000
Anti-mouse HRP	Jackson ImmunoResearch Laboratories	Cat # 111-035-166	1:4000
Antibodies- ELISA			
Anti-mouse IFNγ capture rat mAb Clone R4-6A2	Biolegend	Cat# 505702<br>RRID: AB_315390	2μg/ml
Anti-mouse biotinylated IFNγ capture rat mAb Clone R4-6A2	Biolegend	Cat# 505804<br>RRID: AB_315392	2μg/ml
Antibodies – In Vivo Depletion			
anti-CD4 clone GK1.5 rat mAb	Bio X cell	Cat # BP0003-1 ;<br>RRID: AB_1107636	100μg
anti-CD8 clone 2.43 rat mAb	Bio X cell	Cat # BE0061;<br>RRID: AB_1125541	100μg
anti-Ly6G clone 1A8 rat mAb	Bio X cell	Cat # BP0075-1;<br>RRID: AB_1107721	200μg
InVivoMAb rat IgG2b isotype control clone LTF-2	Bio X cell	Cat # BE0090;<br>RRID: AB_1107780	Match to depletion
Chemicals, Peptides, and Recombinant Proteins			
Normal goat serum	Vector labs	Cat # s-1000	
Triton-X-100	ThermoFisher	Cat # BP151-500	
10% Neutral Buffered Formalin	Fisher chemical	Cat # SF100-4	
Histo-Clear	National Diagnostics	Cat # HS-200	

Fluoroshield Mounting Medium With DAPI	Abcam	Cat # ab104139	
ImmPRESS® HRP Goat Anti-Rabbit IgG Polymer Detection Kit	Vector Labs	Cat # MP-7451	
ImmPRESS® HRP Goat Anti-Rat IgG Polymer Detection Kit	Vector Labs	Cat # MP-7404-50	
ImmPACT® DAB Substrate Kit, Peroxidase (HRP)	Vector Labs	Cat # SK-4105	
M.O.M.® (Mouse on Mouse) Blocking Reagent	Vector Labs	Cat# MKB-2213-1	
ClearMount	StatLab	Cat # MMC0126	
Collagenase A	Sigma Aldrich	Cat # COLLA-RO	
Deoxyribonuclease I	Worthington	Cat # LS002139	
HEPES	Glibco	Cat # 15630080	
Direct Lineage Cell Depletion Kit, mouse	Miltenyi	Cat # 130-110-470	
Chromium Next GEM Single Cell Fixed RNA Sample Preparation Kit	10X Genomics	Cat # PN-1000414	
Software and algorithms			
Fiji (ImageJ)	Commercial	https://imagej.net/software/fiji/	
Prism 10.2.1	GraphPad		
R 4.1.0[56]	R Core Team (2021)	https://www.R-project.org/	
