# Supplementary material for: Respiratory viral infections awaken metastatic breast cancer cells in lungs
Source: Nature. 2025 Jul 30;645(8080):496–506. doi: 10.1038/s41586-025-09332-0 (PMC12422975; doi:10.1038/s41586-025-09332-0)
Supplement: Supplementary file 2 — Reporting Summary [file 41586_2025_9332_MOESM2_ESM.pdf]

nature portfolio

Corresponding author(s): James DeGregori
Last updated by author(s): Nov 24, 2024

Reporting Summary

Nature Portfolio wishes to improve the reproducibility of the work that we publish. This form provides structure for consistency and transparency in reporting. For further information on Nature Portfolio policies, see our Editorial Policies and the Editorial Policy Checklist.

Statistics

For all statistical analyses, confirm that the following items are present in the figure legend, table legend, main text, or Methods section.

n/a	Confirmed	
☐	☒	The exact sample size (*n*) for each experimental group/condition, given as a discrete number and unit of measurement
☐	☒	A statement on whether measurements were taken from distinct samples or whether the same sample was measured repeatedly
☐	☒	The statistical test(s) used AND whether they are one- or two-sided<br>*Only common tests should be described solely by name; describe more complex techniques in the Methods section.*
☐	☒	A description of all covariates tested
☐	☒	A description of any assumptions or corrections, such as tests of normality and adjustment for multiple comparisons
☐	☒	A full description of the statistical parameters including central tendency (e.g. means) or other basic estimates (e.g. regression coefficient) AND variation (e.g. standard deviation) or associated estimates of uncertainty (e.g. confidence intervals)
☐	☒	For null hypothesis testing, the test statistic (e.g. *F*, *t*, *r*) with confidence intervals, effect sizes, degrees of freedom and *P* value noted<br>*Give P values as exact values whenever suitable.*
☒	☐	For Bayesian analysis, information on the choice of priors and Markov chain Monte Carlo settings
☐	☒	For hierarchical and complex designs, identification of the appropriate level for tests and full reporting of outcomes
☐	☒	Estimates of effect sizes (e.g. Cohen's *d*, Pearson's *r*), indicating how they were calculated

Our web collection on statistics for biologists contains articles on many of the points above.

Software and code

Policy information about availability of computer code

Data collection	No software or code was used for data collection.
Data analysis	Data Processing for single cell RNA-seq analysis and bulk RNA-seq analysis is described in Methods, utilizing R (v 4.1.1) and publicly available software Cell Ranger (v 7.1.0), Seurat R package (v 4.3.0), R package scDblFinder (v 1.6.0) (single cell RNA) and limma (v 3.46.0) with the voom method (bulk RNA-seq). Gene set enrichment analysis (GSEA) was performed using the clusterProfiler R package (v 4.0.5). For mitochondrial specific analysis, custom mitochondrial pathway gene lists (Guarnieri et al. Reference provided in manuscript) were utilized, and fGSEA (needs version, don't believe this was a BBSR analysis) was used for pathway analysis using the custom pathway gene lists. For UK Biobank and Flatiron Health data, statistical analyses were conducted using R version 4.1.0. All analyses methods are described in the Methods section, and code for single cell RNA-seq is available on GitHub (https://github.com/Aeg22/dcc_flu).

For manuscripts utilizing custom algorithms or software that are central to the research but not yet described in published literature, software must be made available to editors and reviewers. We strongly encourage code deposition in a community repository (e.g. GitHub). See the Nature Portfolio guidelines for submitting code & software for further information.

Data

Policy information about availability of data

All manuscripts must include a data availability statement. This statement should provide the following information, where applicable:

- Accession codes, unique identifiers, or web links for publicly available datasets
- A description of any restrictions on data availability
- For clinical datasets or third party data, please ensure that the statement adheres to our policy

Data availability - UK Biobank: This study used the UK Biobank data under application number 69328 to MC-H. The UK Biobank received ethical approval from the North West Multi-centre Research Ethics Committee (REC reference: 11/NW/0382) (http://www.ukbiobank.ac.uk/ethics/). UK Biobank data is accessible upon approval from the UK Biobank access committee. Pre-processing/recoding and analytical scripts are available upon request to allow replication of findings by researchers with active UK Biobank access.

Data availability - Flatiron: The data that support the findings of this study were originated by and are the property of Flatiron Health, Inc., which has restrictions prohibiting the authors from making the data set publicly available. Requests for data sharing by license or by permission for the specific purpose of replicating results in this manuscript can be submitted to PublicationsDataAccess@flatiron.com.

Data availability - gene expression: All scRNAseq and bulk RNA-seq data were uploaded to GEO. Raw and processed scRNAseq data is deposited in the Gene Expression Omnibus (GSE264175). For RNA-seq of DCC, raw and processed RNA-seq data are deposited in the Gene Expression Omnibus (GSE282438). Both have been made publicly available, and there will be no restrictions placed on the data.

The bulk RNA-seq was aligned to Ensembl GRCm38, release 102 while the scRNAseq was processed using the Cell Ranger Chromium mouse transcriptome probe set (version 1.0.1).

Research involving human participants, their data, or biological material

Policy information about studies with human participants or human data. See also policy information about sex, gender (identity/presentation), and sexual orientation and race, ethnicity and racism.

Reporting on sex and gender	Electronic health records (EHR) data were analyzed from the UK Biobank and Flatiron Health. For Flatiron Health, as our analyses focused on breast cancer, only biological females were included. For UK Biobank data both sexes were included, of which 53.1% were female. Analyses were sex-matched.
Reporting on race, ethnicity, or other socially relevant groupings	Flatiron EHR data: Age, Gender, Race, Ethnicity, were from the US-based, electronic health record-derived deidentified Flatiron Health Research Database[1]<br>1. Flatiron Health. Database Characterization Guide. Flatiron.com. Published March 18, 2025. Accessed [spelled out Month Day, Year]. https://flatiron.com/database-characterization.<br>UK Biobank data included information on ethnicity, education, employment status, household income which were used for propensity score matching.
Population characteristics	For Flatiron Health, as our analyses focused on breast cancer, only biological females were included. The median age at diagnosis were 59 with IQR (49 to 69). 2.6% were Asian, 12.7% were Black or African American, 9.7% were Other Race, and 75% were White. 92% were Not Hispanic or Latino, and 8% were Hispanic or Latino.<br>For UK BIOBANK at recruitment, the mean age was 60 with IQR (53 - 64) with an average BMI of 27 (IQR 24-30), 53.1% were female, 22.4% had a university degree, 0.9% were Black or African American, 0.7% were Asian, 0.8% Other Race, and 97.6% were White, 43.7% had a higher income. 52.5% was employed.
Recruitment	No active recruitment was carried out. The epidemiological data used for analyses are extracted from existing databases. Inclusion/exclusion criteria for the subjects included in the final analyses were described in the method section.
Ethics oversight	For the Flatiron Health analyses, the Colorado Institutional Review Board approval of the protocol was obtained prior to study conduct and included an informed consent waiver (COMIRB#23-1485, Exemption Category 4). The UK Biobank received ethical approval from the North West Multi-centre Research Ethics Committee (REC reference: 11/NW/0382) (http://www.ukbiobank.ac.uk/ethics/). This information is provided in the Methods section of the manuscript.

Note that full information on the approval of the study protocol must also be provided in the manuscript.

Field-specific reporting

Please select the one below that is the best fit for your research. If you are not sure, read the appropriate sections before making your selection.

☒ Life sciences ☐ Behavioural & social sciences ☐ Ecological, evolutionary & environmental sciences

For a reference copy of the document with all sections, see nature.com/documents/nr-reporting-summary-flat.pdf

Life sciences study design

All studies must disclose on these points even when the disclosure is negative.

Sample size	No sample-size calculation was performed. A sample size of at least 3 samples per group were in each experiment, and each experiment was repeated at least once. Based on previous experience with the metastases models, most experiments were performed with at least samples sizes of 4, as variability in responses to infection were anticipated to necessitate more than the minimal number of 3 for calculations of

	significance.
Data exclusions	No data were excluded from the analyses.
Replication	Each new experiment contains an experimental group for the previous experiment for confirmation of the previous result. All experiments have been successfully replicated at least once in independent experiments.
Randomization	Allocations of animals are randomized. For experiments other than those involving animals, samples were allocated into different treatment groups (e.g. mammospheres treated with vehicle or interleukin-6) in an unbiased fashion (e.g. half the wells get treated with IL-6 and half with vehicle). For in vitro CD8 T-cell killing assays, the different CD8 cell groups were based on the treatment of the donor mice, and then these cells were proportionally split into co-cultures with the different target cells.<br>For in vitro studies, Her2+ organoids were derived from mammary glands of MMTV-Her2 mice of the indicated ages that were randomly selected from our mouse colony for that age and genotype. For EO771 and MET1 cell line studies, no covariates were controlled as all cells were derived from the same source, and thus are considered relatively clonal.
Blinding	Investigators were not blinded in group allocations but were blinded in data collection and data analyses.

Reporting for specific materials, systems and methods

We require information from authors about some types of materials, experimental systems and methods used in many studies. Here, indicate whether each material, system or method listed is relevant to your study. If you are not sure if a list item applies to your research, read the appropriate section before selecting a response.

Materials & experimental systems

n/a	Involved in the study
☐	☒ Antibodies
☐	☒ Eukaryotic cell lines
☒	☐ Palaeontology and archaeology
☐	☒ Animals and other organisms
☒	☐ Clinical data
☒	☐ Dual use research of concern
☒	☐ Plants

Methods

n/a	Involved in the study
☒	☐ ChIP-seq
☐	☒ Flow cytometry
☒	☐ MRI-based neuroimaging

Antibodies

Antibodies used	All information relevant to antibodies are included in the Extended Data Table 1: resource table.
Validation	All antibodies were purchased from commercial sources and validated by the manufacturer, whose websites contain validation statement. Her2 antibody validation data are provided in the manuscript (see Fig 4a). We provide a table (Extended Data Table I) that contains all the antibodies used together with information regarding the manufacturer, catalog number, and dilution; validation for each antibody is described on the manufacturer's web-page. In addition antibody stains were done in parallel with secondary only and with negative controls to insure specificity.

Eukaryotic cell lines

Policy information about cell lines and Sex and Gender in Research

Cell line source(s)	EO771 mammary tumor cells are of C57BL/6 origin, and were the gift of Dr. Diana Cittelly.<br>Vero C1008 (clone E6) were obtained directly from ATCC CRL-1586.
Authentication	For EO771, as a mouse line, STR methods are not available, but we confirmed the expected high expression of p53 and their C57BL/6 origin (as the cells were not rejected in immunocompetent C57BL/6 mice). For Vero cells, the ATCC authenticates all cell lines.
Mycoplasma contamination	EO771 and Vero cells tested negative for mycoplasma.
Commonly misidentified lines (See ICLAC register)	none

Animals and other research organisms

Policy information about studies involving animals; ARRIVE guidelines recommended for reporting animal research, and Sex and Gender in Research

Laboratory animals	Mus musculus FVB female 12-14 weeks old; Mus musculus FVB MMTV-erbB2/neu/HER2 female 12-14 weeks old; Mus musculus FVB MMTV-erbB2/neu/Her2-IL6KO female 12-14 weeks old; Mus musculus C57BL6/J female 14-18 weeks old; Mus musculus C57BL6/J MMTV-erbB2/neu/HER2 female 14-17 weeks old; Mus musculus MMTV-PyMT female 7-9 weeks old; Mus mucsculus MMTV-PyMT-IL6KO female 7-9 weeks old. As described in Methods, all mice were co-housed in specific pathogen free animal facilities, maintained

	at 21 degrees C (+/- 1 degree C), 35% humidity, and a 14 hours light/10 hours dark cycle (6 am-8 pm). All the mice were backcrossed in the C57Bl/6J background for over 10-12 generations. Only female mice were used for the studies, as we are studying metastases derived from mammary gland tumors. The average age of the mice was between 12 to 24 weeks. CO2 followed by cervical dislocation as an approved secondary method was used for euthanasia.
Wild animals	The study did not involved wild animals.
Reporting on sex	Findings from the mouse model applies only to one sex (female).
Field-collected samples	No field collected samples were used in the study
Ethics oversight	University of Colorado Anschutz Medical Campus Institutional Animal Care and Use Committee approved all experiments.

Note that full information on the approval of the study protocol must also be provided in the manuscript.

Plants

Seed stocks	*Report on the source of all seed stocks or other plant material used. If applicable, state the seed stock centre and catalogue number. If plant specimens were collected from the field, describe the collection location, date and sampling procedures.*
Novel plant genotypes	*Describe the methods by which all novel plant genotypes were produced. This includes those generated by transgenic approaches, gene editing, chemical/radiation-based mutagenesis and hybridization. For transgenic lines, describe the transformation method, the number of independent lines analyzed and the generation upon which experiments were performed. For gene-edited lines, describe the editor used, the endogenous sequence targeted for editing, the targeting guide RNA sequence (if applicable) and how the editor was applied.*
Authentication	*Describe any authentication procedures for each seed stock used or novel genotype generated. Describe any experiments used to assess the effect of a mutation and, where applicable, how potential secondary effects (e.g. second site T-DNA insertions, mosiacism, off-target gene editing) were examined.*

Flow Cytometry

Plots

Confirm that:

- [x] The axis labels state the marker and fluorochrome used (e.g. CD4-FITC).
- [x] The axis scales are clearly visible. Include numbers along axes only for bottom left plot of group (a 'group' is an analysis of identical markers).
- [x] All plots are contour plots with outliers or pseudocolor plots.
- [x] A numerical value for number of cells or percentage (with statistics) is provided.

Methodology

Sample preparation	Cells were either recovered from the bronchoalveolar lavage fluid (BALF) by resuspending cell pellets after centrifuging BALF at 500g, 4 degrees C, 5 minutes in PBS with 2%FBS, 2mM EDTA or by digesting whole lungs in collagenase A (Sigma Aldrich cat# COLLA-RO; St. Louis, MO) and deoxyribonuclease I (Worthington cat# LS002139; Lakewood, NJ) with final cells resuspended in PBS with 2% FBS and 2mM EDTA. Cells were strained with 50um cell strainers before antibody staining. For Her2+ sorting, whole lungs were digested using Miltenyi's lung dissociation kit.
Instrument	BD Biosciences LSRII Flow Cytometer, Astrios EQ Flow Cytometer
Software	FlowJo
Cell population abundance	Her2+ cells were 100% within Her2+ sorted fraction as determined by the Astrios EQ Flow Cytometer.
Gating strategy	FSC-A/SSC-A were used to select cells, FSC-A/FSC-H were used to select single cells, live/dead and CD45 were used to select distinct CD45+ cell population. CD4 vs. CD8 axes were used to select CD4+ or CD8+ cells. B220 were used to select B cells, and Ly6G were used to select neutrophils after CD45 gating. CD44, GzmB, FoxP3 were used to select for respective cell populations. Cell types were selected where there are distinct populations (CD45, CD4, CD8, B220, Ly6G), or fluorescent minus one controls (FMO) were used to distinguish positive and negative populations (CD44, FoxP3, GzmB). For Her2+ sorting, Her2+ cells were gated on Her2+CD45- population.

- [x] Tick this box to confirm that a figure exemplifying the gating strategy is provided in the Supplementary Information.
